# Assessment of health promotion content in undergraduate physiotherapy curricula

**DOI:** 10.4102/sajp.v71i1.242

**Published:** 2015-05-29

**Authors:** Kebogile Mokwena, Koketso Phetlhe

**Affiliations:** 1 Department of Social and Behavioural Health Sciences, University of Limpopo, Medunsa Campus (Sefako Makgatho Health Sciences University), South Africa

## Abstract

**Background:**

The integration of health promotion in the treatment of patients should be included in all academic curricula in primary training of health professionals. However, the extent to which health promotion is included in the various curricula at undergraduate level is not known.

**Objective:**

To assess the extent to which health promotion content is integrated in undergraduate physiotherapy training programmes in South Africa.

**Method:**

This was a qualitative and descriptive study, using in-depth interviews with representatives of physiotherapy academic departments.

**Results:**

All universities have some content of health promotion, with the weighting varying between 12% and 40%. Health promotion is taught at various levels of study, and health promotion training blocks are in both urban and rural settings and include communities, schools and old-age homes. The theories of advocacy, enabling and mediation are covered, but there is limited practical training on these elements. There are limited human resources trained in health promotion, as well as a lack of clear processes of developing and reviewing teaching and training materials.

**Conclusion:**

There is lack of consensus on the weighting of health promotion, the level at which it is taught and how it is evaluated across universities. Challenges to integrate health promotion in physiotherapy curricula include lack of frequent curricula reviews, inadequate training of lecturers and lack of conducive practical sites.

The physiotherapy profession needs to reach a consensus on minimum standards for integration of health promotion in undergraduate training, and the physiotherapy professional board has the potential to provide the required leadership.

## Introduction

As global health resources continue to shrink in relation to population growth, the need for and value of the health promotion and disease prevention approach in health services becomes increasingly important. The Ottawa Charter for Health Promotion (World Health Organization 1986) has increasingly become a reference for the development, implementation and evaluation of health promotion programmes (Potvin & Jones 2011). This has identified the need to integrate health promotion into the training curricula of various health disciplines.

The White Paper on the transformation of health in South Africa (South African Government 1997) requires the training of health professionals to shift towards prevention of disease and promotion of health, and not only to focus on curative measures. This was meant to provide a new approach to the organisation of health services in the country (Pillay 2001). However, anecdotal evidence suggests that over the years not much has changed, and that many training programmes have made minimal changes to their curricula. Specifically, Ramklass (2009) stated that physiotherapy curricula have remained static, thus failing to respond to the expectations of the revised South African healthcare system. In addition, Mostert-Wentzel, Frantz and Van Rooijen (2013) identified gaps in preparing physiotherapy undergraduate students for the needs of the South African population and expectations of the Government.

The key principles of the Ottawa Charter include advocacy, an enabling environment and mediation in order to secure prerequisites for basic health. About a decade ago Potts (2006) highlighted the need for health care to focus on the promotion of self-responsibility for health care by patients. The author further challenged health professionals to spearhead the changes through curriculum changes.

Health promotion is universally regarded in the literature as crucial in tackling lifestyle conditions (Dean 2009). The World Health Organization (WHO) defines health promotion as ‘a process of enabling people to increase control over and to improve their health’ (WHO 1986). The role of physiotherapy and health promotion and physical activity has been documented (Foster *et al.* 2005, Trudeau and Shephard 2005; Kallings *et al.* 2008) and there have been calls to further this link between physiotherapy and health promotion in order to combat chronic diseases (Dean 2009).

Although it is assumed that the physiotherapy curriculum often includes some health promotion to enable patients to be independent and manage their health challenges, the extent to which health promotion is formally integrated into the academic programme and the content of health promotion in physiotherapy curricula is not known. A recent study by Walkeden and Walker (2014) highlighted the need to equip physiotherapists with skills to engage in health promotion, as this will enable the profession to meet the challenges of the current epidemic of lifestyle conditions. Therefore the aim of this study was to assess the extent to which health promotion has been integrated in undergraduate physiotherapy curricula in South Africa.

## Research design

### Research approach

This study employed a qualitative design, using in-depth interviews with academic representatives from the eight physiotherapy academic departments in South Africa.

### Research method

#### Population and sampling

The population included academics from the eight physiotherapy training schools in South Africa. The participants were purposively selected by the Heads of Departments of the universities. Of the eight universities approached to participate in the study, seven agreed to participate.

#### Data collection

A letter of invitation was sent to Heads of Departments, requesting them to participate in the study, and to nominate a designated person as a point of contact with the researcher. A quantitative health promotion checklist was developed from the principles of the Ottawa Charter for Health Promotion (WHO 1986) and the guidelines for assessing health promotion programmes (Oldenburg *et al.* 2002; Saunders, Evans and Joshi 2005). The checklist was mailed to all physiotherapy academic departments, who completed it and mailed it back to the researcher. The responses to the checklist were used to develop the interview guide. Upon receiving the checklist the researcher contacted each department telephonically to request an appointment for a face-to-face interview. The interviews were conducted with a designated person in each department, using the self-developed interview guide. Six of the seven interviews were conducted face to face, with one being conducted telephonically. An audio-recorder was used to capture the interviews; each interview took between 45 minutes and an hour.

#### Data analysis

The checklist data were analysed using Epi-Info software, which yielded descriptive statistics of health promotion content that was covered by universities, as well as differences in the training on health promotion.

The audio-recordings were transcribed verbatim and typed into Word documents. A code book with corresponding definitions was developed from the transcripts, and the codes were applied to all of the transcripts using Nvivo 9 software. The analysis identified themes, which were verified by the research supervisor. The themes were used to write the narrative.

#### Trustworthiness

Trustworthiness was achieved by both data and metho­dological triangulation. The interview guide was pre-tested on five qualified physiotherapists, three of whom work in the clinical sphere and two in academic institutions. Data triangulation was applied by collecting data from different sources. Methodological triangulation was applied by using both quantitative (checklist) and qualitative (interviews) data collection methods, and dependability of the findings was enhanced by transcribing the recorded audio qualitative data verbatim.

#### Ethical considerations

Ethical clearance for the study was obtained from the Sefako Makgatho Health Sciences University Research Committee, and permission was obtained from the physiotherapy departments which participated in the study. Informed consent was obtained from all of the interviewees.

## Results

Six of the seven participants were female and they had taught health promotion for periods ranging from one to 7 years, with a mean of 3 years. Although all had some postgraduate qualifications, none of these were in health promotion but were in related fields, including monitoring and evaluation, home-based care rehabilitation and health-risk behaviour.

Seven themes emerged from analysis of the qualitative data: content of health promotion in the curriculum; practical aspects of health promotion training; level at which health promotion is introduced; human resources for teaching health promotion; health promotion aspects that are excluded from the curriculum; assessment of content on health promotion; and challenges to the integration of health promotion in the curriculum.

### Theme 1: Content of health promotion in the curriculum

This theme identified the content of health promotion, which included health promotion strategies and actions from the Ottawa Charter. The content was then contextualised within the role, responsibility and expectations of the physiotherapy profession. All seven universities included some aspects of health promotion, whose focus involved the advocacy role of physiotherapy in the community. Students’ involvement in the community included activities such as health screening, health education, training of caregivers, needs assessment and working with role players. The following quotes illustrate aspects of the health promotion content:

‘We want students to be independent in creativity; students plan their activities from the first day of their block after their orientation. We want them to be able to diagnose, to assess community needs and once they have done that they need to speak to relevant people so that they can address the needs.’ (P7, female)

‘Students are taught to understand the health system and to understand the community needs for health promotion and education within the health system.’ (P6, female)

Applied health promotion activities include educating patients and caregivers about prevention of disease and maintaining a healthy lifestyle, training the patient's family/caregivers to apply prevention measures, training caregivers in the role they could play in the rehabilitation process, identifying role players in communities to assist in interventions, identifying community needs to ensure relevant interventions, rehabilitation of patients within the community, and referral to other health providers. The weighting of health promotion content ranged from an estimated 12% – 40%.

### Theme 2: Practical application of health promotion training

This theme refers to ways in which the theory of health promotion is practically applied in a community setting. The key information that emerged focused on mode of delivery, length of exposure to health promotion setting, activities and content. The respondents indicated that the focus of their teaching was more on health education for individuals and groups, and the integration of health promotion enjoys a minor focus, as illustrated by the following:

‘Students do health promotion at an individual level, e.g. taking tablets and healthy lifestyle, incorporated as part of management. Fourth-year students have a 5 weeks community block, they are the only group that have a practical aspect of which health promotion is highlighted.’ (P2, female)

Students are placed in a community for a period varying from one to 5 weeks to implement health projects, and the practical activities are determined by the communities which students visit. The creation of posters on health education and individual and group therapy provided by the students is commonly practised, and so is conducting health talks to various groups of people with special needs, including stroke victims, the elderly, factory employees, and HIV and/or AIDS patients. The following quote illustrates the practical applications:

‘They must be able to make posters and pamphlets, information leaflets and use tools like overhead projectors if they need to in order to make their presentations in the community, depending on the creativity of the group and the community they are working with. For example, if they are educating teachers they may use laptops or overhead projectors.’ (P1, male)

However, this approach is still limited to clinical diagnosis and is thus limited, because physiotherapy students promote health by providing the knowledge needed for groups and communities to improve their health, without providing the environment that is conducive to healthy choices, and thus do not address all of the determinants of health.

### Theme 3: Level at which health promotion is introduced to students

This theme refers to the year of study in which health promotion is introduced to the physiotherapy curriculum. Whilst the need to introduce health promotion content in first year was espoused, this was lacking in practice. Although limited health promotion activities are promoted at first-year level, this was done without theoretical foundation and is unlikely to ground the students in this aspect.

In practice health promotion theory was mostly introduced in the third year of study, as this level was considered to be appropriate because the students were involved in practical aspects which would help them better understand and integrate health promotion in other physiotherapy practice areas of training. The following quote illustrates this view:

‘It will not be appropriate in first year and they will not understand things like how to teach a patient as they need the foundation of the basic things. In second year there is better understanding and they are able to link things. In first year they still put things in boxes, are not able to link massage and sport injuries. The course is taught in the third quarter of the year (third semester) and at this stage students are more open up as they have already done other physiotherapy courses and they go out to interview patients, they don’t treat as it is for the interview skills and they do health promotion including home programmes. I think that is the logical level.’ (P5, female)

However, one participant was of the view that it would be advantageous for the students to do health promotion at first-year level:

‘I think the students will cope if they had an option of learning health promotion in lower levels; however the university curriculum does not allow.’ (P4, female)

### Theme 4: Human resources for teaching health promotion

This theme refers to the training on health promotion that academic staff received, as well as the number of staff members allocated for the teaching of health promotion.

Human resources for the teaching and promotion of health promotion across the wide curriculum were viewed as inadequate, as most universities had only one lecturer tasked with facilitating the health promotion content part of the curriculum: ‘I am the only person in the department who teaches this course’ (P1, male). This also implies that the content is limited to the views and scope of the particular person. Furthermore, this may disadvantage the selection of health promotion content as well as the development of teaching material.

Two of the universities alternated their staff according to the modules they teach, and do not have designated staff for health promotion integration. Only two institutions were specific on the processes followed to integrate health promotion content into the curriculum. The lack of specific focus on the content suggests that insufficient effort goes into what should be included, particularly in aligning the curriculum with the principles of the Ottawa Charter.

Another limiting factor was the lack of specific training of staff in health promotion, which was illustrated as follows by two participants: ‘I have done a diploma in monitoring and evaluation, not really related to health promotion’ (P7, female) and ‘I have [*postgraduate qualifications*] but not in health promotion, in health-risk behaviour’ (P3, female).

### Theme 5: Health promotion aspects that are excluded from the curriculum

According to the responses training of some health promotion aspects are excluded because of the limitations imposed by the practical areas and the time that students are in the placement although such aspects are covered theoretically: ‘Students are not able to reorient health service as the environment is already provided for them and the focus is on the student understanding how the health system works’ (P5, female).

Another example is the mediation aspect, which could not be conducted by students owing to time constraints and the complexity of the concept: ‘… at that level they can’t do mediation with the people responsible, so they also did education’ (P4, female).

Another reason to exclude the practical aspect of advocacy was that advocacy aims to get the students to influence policy and decision making, and this only comes with experience. Students do not have the skills to fulfil this role, and the aim of the school was to help students understand advocacy at a theoretical level.

### Theme 6: Assessment of content on health promotion

This theme refers to the process of assessing the health promotion content within the physiotherapy curriculum. The assessment of health promotion content is similar across institutions, that is, students are assigned tasks to be completed at the end of the block, which consists of a portfolio on the projects in their block. The portfolio includes their experiences and challenges and how they dealt with these, and is thus also a form of reflection. They present their portfolios in a discussion forum to fellow students and lecturers:

‘In second year they have a theory exam as they do not have a practical aspect. In third year they are tested on theory and they also have an oral presentation on the screening for side-effects of HIV and present scientific evidence for the side effects identified by the students and their role. They also compile a written portfolio using scientific articles on the health promotion programmes that they have conducted in the communities.’ (P4, female)

Two of the institutions have an end of block examination at fourth-year level, which is compulsory for all the students to pass. All the institutions assess health promotion in both the theory and the practical examinations of all physiotherapy subjects, as they are required to integrate this aspect in all the modules. The assessment of health promotion content does not involve external examiners, which may indicate the seriousness attached to health promotion in the curriculum.

### Theme 7: Challenges to the integration of health promotion in the curriculum

This theme refers to what respondents regard as barriers to effective integration of health promotion into the curriculum. These challenges include the lack of qualified physiotherapists in primary health care settings, lack of knowledge of the rehabilitation workers in the same communities, as well as general lack of interest in health promotion on the part of qualified physiotherapists who supervise students in their community work. There is also a view that some physiotherapists in these placements did not always see health promotion as an important aspect of physiotherapy, and in some cases did not even understand the concept, often relying on students to guide them, resulting in demotivation amongst the students. One participant explained the situation as follows:

‘Therapists in the clinical settings do not always see the health promotion content as an essential part of physiotherapy, therefore at times they do not impart adequate knowledge and also they don’t always understand health promotion. I think because of time constraints the physiotherapy curricu­lum as a whole is mostly concentrating on the individual.’ (P7, female)

The length of the community block is also a challenge, because it does not allow adequate time to realise the impact of health promotion: ‘They will not be able to see any results in that period, but it is covered in the theory’ (P3, female).

Logistical challenges include lack of adequate accom­modation, which often means that health promotion practice is limited to areas close to the university. The inadequate and inconsistent integration of health promotion in other physiotherapy subjects was raised as a concern. Also identified is students’ initial lack of interest in health promotion – but this seems to improve with time for all groups of students: ‘… on the students’ side, their attitude becomes more positive as they get more exposure to the practical aspect. In their reflective diary they note challenges at the beginning [*of the block*]’ (P4, female).

## Discussion

Physiotherapy is in itself therapy for already diseased people, and creativity in applying the principles of health promotion requires grounded understanding of health promotion principles as well as academic creativity to enable the application of these principles to physiotherapy practice and student training. The finding that health promotion content is mainly limited to health education confirms the findings of Perreault (2008), who stated that health education is the most used health promotion strategy in physiotherapy practice. This identifies the need for the physiotherapy profession – specifically academia – to extend their teaching and application of health promotion to include other elements of health promotion, specifically patient empowerment, which is deemed to be a central concept in health promotion (Walkeden & Walker 2014).

The theory of the Ottawa Charter principles of advocacy, mediation, enablement, strengthening community action, and developing personal skills are satisfactorily covered by some institutions. However, the findings include inadequate training in the practical aspects of health promotion, which are essential for the full spectrum of health promotion practice. The limitation may be associated with the training platform utilised in physiotherapy practice, which mainly focuses on clinical aspects of health, with a less prominent role for the promotion of health outside the clinical arena. However, there is growing pressure for emphasis on health promotion in hospitals (Walkeden & Walker 2014), which requires physiotherapy academics to teach and apply the principles accordingly.

According to Perreault (2008) there are differences in the conceptualisation of health between health promotion and physiotherapy, and these differences need to be acknowledged and addressed to enable the full integration of health promotion into the physiotherapy curriculum. The findings of this study suggest that such integration has not yet fully occurred, and thus renders the training content insufficient as it deviates from the principles of the Ottawa Charter. The inclusion of these elements in both theory and practice must be part of the focus of the content on health promotion.

### Recommendations

The following recommendations emerged from the findings of the study:

- That the physiotherapy profession in South Africa dis­cusses and reaches consensus on minimum re­quirements for the inclusion of health promotion in undergraduate curricula, as well as providing guidelines on implementation and developing strategies to evaluate health promotion content.
- That physiotherapy academic departments invest in human resources for the teaching of health promotion, as this may extend from integration of health promotion in training to overall physiotherapy practice.

## Conclusion

The health promotion content of physiotherapy curricula seems to be less than that contained in the Ottawa Charter for health promotion. Some activities that students are involved in do indicate that they are capable of playing their role in promoting health; however, it seems that in many instances students are not aware of that role, and are limited by the time allocated by the health promotion block as well as the teaching and training platform for physiotherapy students. The lack of guidance from regulatory bodies such as the Health Professions Council of South Africa and the Physiotherapy Society might also be contributing to these gaps.
